# Health Literacy and Patient Empowerment: Separating Con-joined Twins in the Context of Chronic Low Back Pain

**DOI:** 10.1371/journal.pone.0118032

**Published:** 2015-02-13

**Authors:** Anne-Linda Camerini, Peter J. Schulz

**Affiliations:** Institute of Communication and Health, Università della Svizzera italiana, Lugano, Switzerland; Catholic University of Sacro Cuore, ITALY

## Abstract

**Objectives:**

While health literacy has been widely considered key to patient empowerment, an alternative approach separates both concepts and distinguishes between dif-ferent types of patients according to their levels of health literacy and empowerment. These types are deemed to vary in their health-related actions and outcomes. In this study, we exam-ine the relationship between health literacy and patient empowerment and compare socio-demographic characteristics, health-related activities, and health outcomes in four types of pa-tients suffering from chronic low back pain (cLBP).

**Methods:**

In a cross-sectional study, 273 cLBP patients from four Swiss can-tons (Vaud, Geneva, Fribourg, Ticino) and Lombardy (Italy) were invited by their healthcare providers to complete a self-administered paper-and-pencil questionnaire which assessed pa-tients’ health literacy, empowerment, involvement in the medical encounter, medication non-adherence, and perceived pain and functionality as a measure of health outcomes.

**Results:**

Health literacy and patient empowerment were not significantly correlated with each other, *r*(271) = .09, *p* > .05, allowing to differentiate be-tween four types of patients based on their levels of health literacy and patient empowerment. Subsequent chi-square tests and analyses of variances revealed significant differences among patients that could, however, only be attributed to health literacy, as in the case of age and ed-ucational attainment, or patient empowerment, as in the case of patients’ involvement in the medical encounter. No significant differences were evident for gender, medication non-adherence, and health outcomes.

**Conclusion:**

The study provides empirical evidence for the need to consider health literacy and patient empowerment as independent concepts in the context of cLBP but calls for further studies to be able to conclude on how the two concepts interact and determine health-related activities and outcomes.

## Introduction

The prevalence of chronic low back pain (cLBP) is rising [1] with significant impact on the individual and societal level: on the individual level, cLBP causes physical impairment and psychological distress reducing patients’ and their families’ quality of life [2, 3]. On the societal level, the disease is associated with considerable direct (use of medication and healthcare services) and indirect (productivity losses due to sick leaves) costs [4–6]. Effective therapies of cLBP include multimodal treatments with pharmacological, physical, and psychological actions requiring patients to take an active role in the management of their disease [7]. Along with this active role comes the need to have both the knowledge and skills as well as the intrinsic motivation to effectively manage the disease. While knowledge and skills are attributed to patients’ health literacy, motivation is associated with patient empowerment [8]. Health literacy and patient empowerment are considered key concepts in the health context and more so in the context of chronic disease management [9–11]. Before we examine the relationship between health literacy and patient empowerment and their significance in the context of cLBP, we briefly introduce both concepts and illustrate conflicting views on how they are related to each other and thought to determine health-related decisions and actions.

### Health Literacy

Health literacy in its original sense is defined as a “constellation of skills, including the ability to perform basic reading and numeric skills required to function in the health care environment” [12]. It has soon been considered a multidimensional concept including more advanced skills to obtain, process, and understand basic health information and services and to critically evaluate them in light of one’s personal situation and needs [13–16]. As such, health literacy comprises the ability to perform knowledge-based tasks specific to different health contexts [17]. In the context of cLBP, knowledge-based tasks are particularly critical to self-management activities and require both declarative and procedural knowledge [18]. Declarative knowledge is any factual knowledge related to cLBP that is needed to learn how to approach the disease. On the other hand, procedural knowledge is the “know-how” to apply factual knowledge and use it effectively in the management of the disease. The acquisition of declarative and, to a smaller extent, procedural knowledge oftentimes requires functional health literacy skills. However, these skills are not essential qualifying health knowledge as an informative dimension in the broader concept of health literacy [19].

### Patient Empowerment

When talking about empowered patients, empowerment, is “[…] characterized by perceptions of control regarding one’s own health and health care; perceptions of competence regarding one’s ability to maintain good health and manage interactions with the health care system; and internalization of health ideals and goals” [20]. As shown in this citation, patient empowerment is a multidimensional concept that has rarely been explicitly measured but regularly invoked as a factor in the success of empowerment interventions [8]. Schulz and Nakamoto [8] define patient empowerment as an umbrella term encompassing four distinct dimensions borrowed from the management literature [21] and adopted to the health context. These dimensions include *meaning* or the degree to which patients think that what they do with respect to their health is meaningful and important, *competence* or the degree to which patients feel competent to perform self-management activities, *self-determination* or the degree to which patients think that what they do about their health is determined by themselves, and *impact* or the degree to which patients feel that self-management activities make a difference in their health status. All four dimensions are considered volitional in nature, motivating patients to take action with regards to their health.

### Health Literacy and Patient Empowerment: Conjoined Twins?

**Fig 1:**
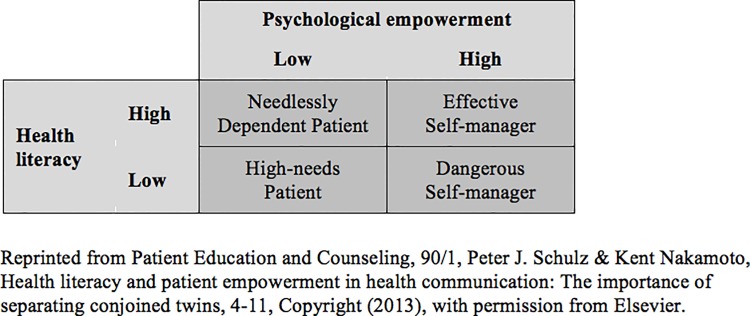
Health literacy, psychological empowerment, and patient behavior.

While often cited definitions of health literacy and frameworks incorporating the concept claim that health literacy empowers patients to take action with positive effects on their health [22–25], more recent voices argue that patient empowerment is different from health literacy as the latter focuses on the knowledge and skills needed to take action but is not itself necessarily motivating [8, 26]. These voices challenge the understanding of health literacy as a necessary condition of patient empowerment as they separate both concepts and consider health literacy and patient empowerment as independent yet equally important factors that determine health-related decisions and actions. With this in mind, Schulz and Nakamoto [8] propose a two-by-two matrix identifying four different types of patients according to their levels of health literacy and psychological (patient) empowerment (Fig. 1).

The four types of patients are thought to act differently dependent on whether they are high or low in their health literacy and patient empowerment: *High-needs patients* lack both the knowledge and skills as well as feelings of being empowered hindering them to make appropriate decisions for their health. *Needlessly dependent patients* may choose to be highly dependent on healthcare providers despite their ability to make well informed decisions for their health incurring needless costs. On the contrary, *dangerous self-managers* are empowered patients who assume an authoritative role in their healthcare decisions lacking adequate knowledge and skill and who could, thus, make dangerous choices that impede their health goals. *Effective self-managers* use healthcare resources appropriately to optimize their health outcomes. In contrast to *effective self-managers*, who are high in health literacy and empowerment, *high-needs patients*, *needlessly dependent patients*, and *dangerous self-managers* are deemed to pose problems not only for themselves but also for the healthcare system as they lack the skills and knowledge and/or the motivation to engage in constructive health-related activities.

### Study Objectives and Hypotheses

The patient classification just outlined is rather conceptual in nature. We, therefore, aim at empirically testing the existence of and associated differences in the four types of patients with regards to their socio-demographic characteristics and their health-related activities and outcomes. We do so in the context of a specific chronic disease, namely cLBP. If the proposed patient classification holds true, our results would not only contribute to the initiated debate on the interrelation between health literacy and patient empowerment, but also provide practical implications for healthcare providers. Indeed, a better understanding of how patients’ levels of health literacy and empowerment impact their actions in a specific health context like cLBP helps to adequately address patients’ needs and contribute to positive health outcomes.

As part of this study we first ask:

RQ1: Is the classification of patients according to the two-by-two matrix proposed by Schulz and Nakamoto [8] meaningful among cLBP patients?

To make the classification meaningful, health literacy and patient empowerment need to be sufficiently different from each other. Thus, we hypothesize that there is no significant correlation between health literacy and patient empowerment (H1).

If all four types of patients exist, we subsequently ask:

RQ2: How do the four types of patients differ according to a) their socio-demographic characteristics, b) their health-related activities, and c) their health outcomes with regards to cLBP?

Due to the exploratory nature of this study, a-priori hypotheses about differences in the socio-demographic characteristics are difficult to formulate, especially with regards to gender. However, based on findings from previous health literacy studies, one can expect that patients with low health literacy—independent of their level of empowerment—tend to be older (H2) and less educated (H3) than patients with high health literacy [27, 28].

When it comes to health-related activities in the context of cLBP, we can hypothesize differences in patients’ involvement in their treatment plan as well as in their adherence to recommended self-management activities including medication intake. We expect that both the dangerous self-manager and the effective self-manager, sharing high levels of patient empowerment, tend to engage more with their healthcare provider by specifically asking for information in the doctor-patient encounter than the other two types of patients (H4). The translation of acquired information into constructive self-management activities may, however, differ as we expect that patients with low health literacy and high empowerment (dangerous self-managers) tend to adhere less often to their prescribed medication regimen than patients with high health literacy and high empowerment (effective self-managers) (H5). Considering medication non-adherence as a function of low health literacy [29]—independent of patient empowerment—we furthermore expect no differences in reported medication non-adherence between the dangerous self-manager and the high-needs patient, who both share low levels of health literacy (H6).

Positive health outcomes in the context of cLBP are attributed to reduced pain and improved functionality. These are hypothesized to occur more frequently among effective self-managers than the other three patient types as a result of appropriate self-management activities (H7).

## Methods

### Procedure

To address our research questions and hypotheses, we collected data from 273 cLBP patients during a cross-sectional study between January 2012 and 2013 in Italian-speaking Lombardy (Italy) and Canton Ticino (Switzerland) as well as in the French-speaking Cantons Vaud, Geneva, and Fribourg (Switzerland). Both outpatients and inpatients were recruited through their healthcare providers. Healthcare providers were specialists in rheumatology, physiotherapy, pharmacology, and neurosurgery affiliated with hospitals or working in medical practices. Patients were eligible for participation in the study if they were aged 18 or older, if they had suffered from cLBP for at least three months, if their pain was not caused by cancer, systematic inflammatory disease, or fibromyalgia syndrome (FMS), and if they had sufficient knowledge of Italian and French respectively. Patients were asked to complete a self-administered paper-and-pencil questionnaire. An assistant was present to clarify eventual comprehension problems. After completion of the questionnaire, patients recruited in Switzerland received a small incentive in the amount of ten Swiss Francs. In Lombardy, respecting common research practices, no incentive was given.

### Ethics Statement

The Cantonal Ethics Committees of Vaud (Commission cantonale d’éthique de la recherche sur l’être humain du canton de Vaud), Ticino (Comitato etico cantonale Ticino), and Fribourg (Commission d’éthique de recherche du canton de Fribourg) as well as the Institutional Review Boards of the collaborating hospitals in Geneva (Commission centrale d'ethique des hôpitaux universitaires de Genève), and Lombardy (Comitato etico dell’azienda ospedaliera istituto ortopedico Gaetano Pini di Milano) approved this study. Before completing the questionnaire, all patients gave their written informed consent to publish their responses in anonymized and aggregated form.

### Measures

The self-administered paper-and-pencil questionnaire contained measures based on patients’ self-reports. Since none of the measures was previously translated and validated in Italian or French, the questionnaire was translated and back-translated by two independent bilingual translators for each language (English/Italian and English/French) to assure linguistic validity [30, 31]. Prior to data collection, the questionnaire was pre-tested in five cLBP patients and assessed by one healthcare provider for face and content validity, again for each language separately. Besides measures for socio-demographic characteristics such as gender, age, and highest educational attainment of patients, the questionnaire included measures for the following concepts:

*Health literacy* was operationalized as declarative and procedural knowledge assessed by twelve questions from the Low Back Pain Knowledge Questionnaire [32] and based on information from cLBP websites (S1 Materials). Questions addressed knowledge related to symptoms, causes, treatments, and the management of cLBP. Each question (e.g., “What is chronic low back pain?”) was followed by a set of four response possibilities and an “I don’t know” option. Correct responses were coded as 1, incorrect and “I don’t know” responses as 0. The final measure was obtained by a mean score calculation with a theoretical range from 0 (no correct response) to 1 (all correct responses) (*M* = 0.63, *SD* = 0.20, *Median* = 0.63)

*Patient empowerment* was measured with the Psychological Empowerment Scale developed and validated by Spreitzer [33] for the use in workplace settings. Incorporating the multidimensionality of the concept, the scale consists of three items for each of the four sub-dimensions adapted to the context of cLBP and its management (File S1): meaning (e.g., “Dealing with my back pain is very important to me.”), competence (e.g., “I am confident about my ability to do deal with my back pain.”), self-determination (e.g., “I have significant autonomy in determining how I deal with my back pain.”), and impact (e.g., “I have a great deal of control over the management of my back pain.”). Patients responded on a Likert scale ranging from 1 (strongly disagree) to 7 (strongly agree) with higher values suggesting higher levels of empowerment. Troublesome skewness and kurtosis values were observed for meaning indicating non-normality and lack of variance. Thus, the sub-dimension was dropped and a mean score calculated for the remaining nine items (*M* = 4.71, *SD* = 1.25, *Median* = 4.78). The scale showed good internal consistency (*α* = .88).

*Patient involvement* in their treatment plan was assessed with four items from the patient information provision subscale as part of the modified version of the Patients’ Perceived Involvement in Care Scale (M-PICS) [34]. Patients responded to these items (e.g. “I ask my physician a lot of questions about my chronic low back pain.”) on a 5-point scale ranging from 1 (never) to 5 (always) with higher values indicating higher levels of patient involvement. The subscale showed good internal consistency (*α* = .90) and a mean score was calculated for further analyses (*M* = 3.43, *SD* = 1.20).

*Medication non-adherence* was used as an indicator for detrimental self-management activities in the context of cLBP. It was measured with twelve 5-point Likert items from the Pain Medication Questionnaire [35] (e.g., “I get pain medication from more than one doctor in order to have enough medication for my pain”). Three items were reversely formulated and recoded before calculating a mean score that ranged from 1 (always adhering to medication regimen) to 5 (never adhering to medication regimen) (*M* = 1.79, *SD* = 0.63). The scale showed acceptable internal consistency (*α* = .74).

*Health outcomes* were measured with six items from the Chronic Pain Grading Scale [36]. Three items measured pain intensity (e.g., “In the past 3 months, on average, how intense was your pain?”) on an 11-point scale ranging from 0 (no pain) to 10 (pain as bad as it could be). Another three items measured functionality (e.g., “In the past 3 months, how much has this pain interfered with your daily activities (e.g. getting dressed, doing shopping)?”) on an 11-point scale ranging from 0 (no interference of pain/no change) to 10 (unable to carry on activities/extreme change). Lower values imply better health outcomes. The scale with all six items showed good internal consistency (*α* = .89), and a mean score was calculated for further analyses (*M* = 5.42, *SD* = 2.14).

### Sample

The sample consisted of 153 cLBP patients from the French-speaking cantons, 53 from Italian-speaking Canton Ticino, and 67 from Lombardy. To attain a diverse cLBP population, both outpatients (216) and inpatients (57) were recruited. With regards to their socio-demographic characteristics, 159 patients were female (58%). Patients ranged in age from 20 to 89 years (*M* = 50.70, *SD* = 13.94). One hundred and eighty three (67%) held an educational degree beyond obligatory school, which corresponds to ten years of school in Switzerland and eight years of school in Italy. Of these patients, the majority (145) reported to have a post-secondary non tertiary educational degree and 38 patients a university degree. Among the remaining ninety patients (33%), 41 patients held no degree or finished primary school while 49 patients reported to have a secondary school degree. On average, patients had suffered from low back pain for ten years (*M* = 10.0, *SD* = 10.2).

## Results

### Preliminary Analyses

Data were analyzed in SPSS © 21. In preparation of primary data analyses, we examined the data for non-normality, outliers, and missing values. Troublesome skewness and kurtosis values were observed for one sub-dimension of patient empowerment, which—as previously described—was excluded from primary analysis. We furthermore evaluated the data for outliers at the univariate level by examining absolute values greater than three standard deviations from the mean. A minimal number of univariate outliers was detected and, therefore, retained. Missing data analysis revealed less than 10 percent missing values for each item. To check whether data were missing at random, a dummy variable was created for each item with 1 “presence of missing values” and 0 “absence of missing values.” When correlating these dummy variables with socio-demographics, no significant coefficients were found. Eventually, we imputed values for missing data based on an Expectation Maximization algorithm implemented in the statistical software package.

### Primary Analyses

To empirically test the existence of the four types of patients classified according to their levels of health literacy and empowerment, we conducted a bivariate correlation analysis. No significant correlation was found between health literacy and patient empowerment, *r*(271) = .09, *p* > .05, indicating that the two concepts are indeed distinct from each other allowing to differentiate between four types of patients. Hence, H1 was confirmed.

To be able to compare these types of patients, a median split was performed for health literacy (*Median* = 0.63 on a scale from 0 to 1) and patient empowerment (*Median* = 4.78 on a scale from 1 to 7) to produce roughly equally sized groups. As a result, we identified 77 patients with low health literacy and low patient empowerment (high-needs patients), 71 patients with high health literacy and high patient empowerment (effective self-mangers), 61 patients with low health literacy and high patient empowerment (dangerous self-managers), and 64 patients with high health literacy and low patient empowerment (needlessly dependent patients).

Next, we performed chi-square tests and analyses of variance (Table 1) with subsequent Tukey’s post hoc tests (Table 2) to understand if and how these four types of patients differ according to their socio-demographic characteristics, selected cLBP management activities, and health outcomes.

**Table 1:** Summary of results overall and by patient type.

	Overall (n = 273)	High-needs Patient (HNP) (n = 77)	Needlessly Dependent Patient (NDP) (n = 64)	Dangerous Self-manager (DSM) (n = 61)	Effective Self-manager (ESM) (n = 71)	Significance level
Socio-demographics						
Gender (female) ^a^	159 (58)	46 (17)	36 (13)	30 (11)	47 (17)	.253
Age ^b^	50.70 (13.94)	55.07 (15.65)	46.52 (11.71)	53.47 (14.64)	47.34 (11.27)	< .001***
Education (> obligatory school) ^a^	183 (67)	38 (14)	51 (19)	39 (14)	55 (20)	< .001***
Health-related activities						
Involvement in medical encounter ^b^	3.43 (1.20)	3.25 (1.20)	3.19 (1.02)	3.81 (1.29)	3.52 (1.20)	.012*
Medication non-adherence ^b^	1.79 (0.63)	1.89 (0.69)	1.87 (0.68)	1.68 (0.54)	1.71 (0.57)	.100
Health outcomes						
cLBP pain and functionality ^b^	5.42 (2.14)	5.04 (2.01)	5.39 (1.75)	5.43 (2.10)	5.81 (2.53)	.187

Note: Values are based on data with imputed missings following an expectation maximization approach

^a^ *n* (%) and Chi-square statistics

^b^ *M* (*SD*) and F-statistics

*p < .05,

**p < .01,

***p < .001.

**Table 2:** Results of Tukey’s post hoc tests.

	Mean difference between HNP and NDP	Mean difference between HNP and DSM	Mean difference between HNP and ESM	Mean difference between NDP and DSM	Mean difference between NDP and ESM	Mean difference between DSM and ESM
Socio-demographics						
Age	8.55*	1.60	7.73*	-6.95*	-0.82	6.13*
Health-related activities						
Involvement in medical encounter	0.06	-0.56*	-0.27	-0.62*	-0.33	0.29
Medication non-adherence	0.02	0.21	0.18	0.19	0.16	-0.03
Health outcomes						
cLBP pain and functionality	-0.35	-0.39	-0.77	-0.04	-0.42	-0.38

Note: Values are based on data with imputed missings following an expectation maximization approach

Values indicate mean differences between patient types

*p < .05

**p < .01

***p < .001.

With regards to gender, no a-priory hypothesis was formulated. However, Pearson’s chi-square test showed that all four types of patients did not significantly differ by gender, chi-square (3, *N* = 273) = 4.08, *p* = > .05. As for patients’ age and educational attainment, we expected that high-needs patients and dangerous self-managers, characterized by low levels of health literacy, were older and less educated. Conducting analysis of variance, we found a significant difference for age, *F*(3, 273) = 7.06, *p* = < .001. Tukey’s post hoc test revealed that patients with low health literacy were significantly older than patients with high health literacy—independent of their level of psychological empowerment—but they did not significantly differ from each other. Thus, H2 was confirmed. Patient types also differed by their educational attainment, chi-square (3, *N* = 273) = 19.27, *p* = < .001. The examination of adjusted residuals revealed that study participants belonging to the types of patients characterized by a high level of health literacy (needlessly dependent patient and effective self-manager) had significantly more often an educational degree beyond obligatory school. On the contrary, study participants belonging to the types of patients characterized by a low level of health literacy had less often an educational degree beyond obligatory school. But this was only significant for patients low in health literacy and empowerment (high-needs patient). Thus, H3 was partly confirmed.

With regards to health-related activities, we hypothesized that highly empowered patients—independent of their health literacy level—were more engaged in the medical encounter (i.e requesting more information from their healthcare providers) than patients with low empowerment. Analysis of variance showed a significant difference, *F*(3, 273) = 3.72, *p* = < .05. Tukey’s post hoc test revealed that dangerous self-managers, who are high in patient empowerment but low in health literacy, significantly stronger agreed to asking their healthcare provider for information about their treatment plan (*M* = 3.8) than high-needs patients (*M* = 3.3) and needlessly dependent patients (*M* = 3.2). No significant difference was evident when the dangerous was compared to the effective self-manager (M = 3.5). Thus, H4 was confirmed. We furthermore hypothesized that dangerous self-managers tend to adhere to their medication regimen less often than effective self-managers, attributing non-adherence to limited health literacy. However, the difference in self-reported medication non-adherence among all types of patients was not significant, *F*(3, 273) = 2.11, *p* = > .05. Given that, H5 was not confirmed. At the same time, H6 was confirmed stating that patients with low health literacy, independent of their level of empowerment, do not significantly difference in their self-reported medication non-adherence.

Lastly, we wanted to see whether the four types of patients differed in their health outcomes operationalized as perceived pain and functionality. We expected that effective self-managers have better health outcomes as a result of appropriate self-management activities. Analysis of variance showed no significant difference, *F*(3, 273) = 1.61, *p* = > .05, although the tendency is as expected: Effective self-managers on average reported to have less pain and to be less limited in their everyday functionality (*M* = 5.0) than dangerous self-managers and needlessly dependent patients (both *M* = 5.4) as well as high-needs patients (*M* = 5.8). Nevertheless, H7 was not confirmed.

## Discussion

The present study builds on theoretical advancements concerning the conceptualization of health literacy and patient empowerment and on how these concepts are related to each other. We aimed to test the proposed distinction of the concepts by considering health literacy and patient empowerment as two independent yet equally important determinants of health-related activities and health outcomes [8]. Applied to the context of a specific chronic disease, i.e. cLBP, the lack of a significant correlation between health literacy and patient empowerment supported the assumed independency between the two concepts challenging the widely shared understanding of health literacy as an empowering tool [22–25]. We subsequently classified patients into four different types based on their levels of health literacy and patient empowerment. These types differed from each other not only in their socio-demographic characteristics, but also in their activities related to the management of cLBP and health outcomes. However, significant differences could only be attributed to health literacy, as in the case of age and educational attainment, *or* patient empowerment, as in the case of patients’ involvement in the doctor-patient encounter. No significant differences were evident for gender, medication non-adherence, and health outcomes. Given these results, health literacy and patient empowerment do not interact when associating these concepts with socio-demographic characteristics and health-related activities. Especially with regards to health-related activities and outcomes, the question remains whether this finding is specific to the sample of cLBP patients and measures at hand, or whether it holds true across various health contexts and patient populations. Thus, further empirical studies are needed to evaluate the applicability of Schulz and Nakamoto’s [8] conceptual patient classification.

Drawing our attention to the significant differences between patients with low and high empowerment with regards to their self-reported involvement during the medical encounter, we can conclude that patients with low empowerment show a preference for what is termed the “paternalistic approach” in the doctor-patient-relationship as described by Roter and Hall [37]. As patients with low empowerment do not engage in the medical encounter, they leave the medical expertise and decisions to their healthcare provider. However, we think that whereas needlessly dependent patients *want* their healthcare provider to assume the role of guardians, high-needs patients *need* their healthcare provider to assume this role as they not only lack the intrinsic motivation to become involved in their treatment plan but also the necessary knowledge and skills to do so. On the contrary, patients with high empowerment seem to prefer a patient-centered approach where the doctor-patient-relationship can either be characterized by mutuality or consumerism [37]. One can expect that effective self-managers prefer a mutual relationship with healthcare providers negotiating their treatment plan, whereas dangerous self-managers may more likely act as consumers challenging the advisory role of healthcare providers as these patients lack the knowledge and skills to engage in a fruitful mutual relationship. Our significant findings with regards to differences in cLBP patients’ involvement in the medical encounter as a function of perceived empowerment are a good starting point to confront different types of patients with different styles of doctor-patient communication [38, 39]. But further studies using refined measures and accounting for healthcare providers’ characteristics are needed to draw theoretically and empirically grounded conclusions.

## Study Limitations

Some methodological limitations of this study must be acknowledged. Firstly, the results of this study are based on a convenient sample recruited through a limited number of collaborating healthcare providers who were not required to follow specific randomization techniques during the recruitment process. Due to financial and temporal constrains, the sample was also rather small and we were not able to detect potential differences in, e.g., health outcomes among the four different types of patients. Secondly, when measuring health literacy, we did not include more advanced skills linked to critical health literacy and judgment skills. This limitation resides in the fact that to date objective measures of these more advanced skills in the context of cLBP are missing. First attempts to assess judgment skills with objective measurement tools have been made in other chronic disease contexts, e.g., insomnia [40] and asthma [41], providing a good starting point to develop and validate such tools for their eventual use in the context of cLBP. Thirdly and lastly, medication non-adherences was assessed with self-report measures. Although these measures were previously validated in the context of chronic pain, they did not allow to detect significant differences in medication non-adherence in our sample of cLBP patients. This is presumably due to potential social desirability effects as a consequence of the recruitment process: Patients recruited through their healthcare providers might have either accepted to participate in the study because they have a good relationship with their healthcare provider and therefore tend to adhere to prescribed medical regimens or they might have avoided to admit medication non-adherence in fear of possible consequences for the relationship with their healthcare providers.

### Theoretical and Practice Implications

Contrary to the widely shared assumption that health literacy leads to increased patient empowerment, this study provides empirical evidence for the need to separate both concepts and to consider health literacy and patient empowerment as independent determinants in the context of cLBP and its management. This finding has both theoretical and practice implications. With regards to the theory, we hope to have triggered the study of these concepts and their interrelation in different health contexts and study populations to eventually contribute to an alternative way of theorizing health literacy and patient empowerment and their role in the health context. With regards to practice, we hope to have raised the awareness of healthcare providers for the need to consider and address patients’ levels of health literacy and empowerment to be able to support them in the management of their disease and, thus, contribute to positive health outcomes. This can be achieved through targeted patient education providing easy to understand information on the treatment and management of chronic diseases like cLBP. In addition, a patient-centered environment where patients can raise concerns and participate in decisions regarding their treatment improves patient empowerment and their willingness to engage in health-related activities. It is, however, important to acknowledge that there is no one-fits-all-solution as we have given first empirical evidence on the need to separate health literacy and patient empowerment as presumably conjoined twins.

## Supporting Information

S1 Materials Measures of health literacy and patient empowerment. (DOCX)

S1 Dataset. (SAV)
