# Supplementary material for: Health Literacy and Patient Empowerment: Separating Con-joined Twins in the Context of Chronic Low Back Pain
Source: PLoS One. 2015 Feb 13;10(2):e0118032. doi: 10.1371/journal.pone.0118032 (PMC4332654; doi:10.1371/journal.pone.0118032)
Supplement: S1 Materials — (DOCX) [file pone.0118032.s001.docx]

*Measures of health literacy (declarative and procedural knowledge)*

*Taken from the Low Back Pain Knowledge Questionnaire (Maciel et al., 2009) and information on cLBP websites, multiple-choice questions with one or two true answers per question as indicated*

What is chronic low back pain? Mark ONE correct alternative:

Pain in the lumbar region that usually improves in three weeks, with or without treatment

Untreatable pain in the lumbar region

Pain in the lumbar region requiring surgery

Pain in the lumbar region lasting more than 3 months *(True)*

I don’t know

These can cause low back pain. Mark TWO correct alternatives:

Cold and aging

Postural problems, arthrosis and a herniated disc *(True)*

Tumors, infections and fractures *(True)*

Diabetes

I don’t know

These are symptoms of low back pain. Mark TWO correct alternatives:

A cough, sluggishness and loss of energy

Tiredness and pain throughout the body

Pain in the lumbar region that worsens when carrying weight *(True)*

Difficulty in picking up objects from the floor *(True)*

I don’t know

The highest incidence of back pain is between what ages? Mark ONE correct alternative:

20-34 years old

35-55 years old *(True)*

Over 55 years old

Equal among all age groups

I don’t know

What are the most common symptoms of a herniated disc? Mark TWO correct alternatives:

Sciatica – a sharp pain that runs from buttocks down one leg *(True)*

Burning pain in the neck

Shooting back pain when you cough *(True)*

Burning pain in both arms

I don’t know

In regards to drug treatment for low back pain, mark TWO correct alternatives:

Anti-inflammatory medicines and analgesics may be used during acute crises *(True)*

Corticosteroids are not recommended during an acute crisis

Antidepressants and anticonvulsants may be used for chronic low back pain *(True)*

Topical medications such as gel, plasters or ointments are always indicated

I don’t know

In regards to physical activity and low back pain, mark TWO correct alternatives:

Walking three times a week for an hour can worsen chronic low back pain

Intensive exercises are indicated for acute low back pain

Aquatic activities may be beneficial to the patient with chronic low back pain *(True)*

The most highly recommended exercises are strengthening of the abdomen and the back mucles, stretching and physical conditioning *(True)*

I don’t know

To protect the spine, mark TWO correct alternatives:

Wear high heels all day

Sit down to put on your socks and shoes *(True)*

Carry weight on one side of the body

Wash the dishes with your stomach leaning against the sink *(True)*

I don’t know

What is best for your back when you sleep? Mark ONE correct alternative:

On your back

On your stomach

On one side

On one side with the knees flexed toward the chest *(True)*

I don’t know

In regards to surgical treatment for low back pain, mark TWO correct alternatives:

It is indicated in few cases *(True)*

It may be important in cases with nerve root compression and spinal column instability that do not improve with clinical treatment *(True)*

Surgery guarantees the cure of low back pain

It is the best treatment for any type of low back pain

I don’t know

In regards to prolonged bed rest, mark TWO correct alternatives:

It is the primary recommended treatment for low back pain

It is not the recommended treatment for low back pain *(True)*

It worsens low back pain *(True)*

It has no negative effects on low back pain

I don’t know

How many adults will experience lower back pain at some time in their lives? Mark ONE correct alternative:

2 out of 10

4 out of 10

6 out of 10

8 out of 10 *(True)*

I don’t know

*Measures of patient empowerment*

*Adapted from the Psychological Empowerment Scale (Spreitzer, 1995). Items are measured on a 7-point Likert-scale ranging from 1 “strongly disagree” to 7 “strongly agree”*

*Meaning*

Dealing with my back pain is very important to me.

The activities I do to handle my back pain are meaningful to me.

Dealing actively with my back pain is meaningful to me.

*Competence*

I am confident about my ability to do deal with back pain.

I am self-assured about my capabilities to deal with back pain.

I am prepared to do the activities necessary to handle my back pain.

*Self-determination*

I have significant autonomy in determining how I deal with my back pain.

I can decide on my own how to handle my back pain.

I have considerable opportunity for independence and freedom in how I deal with back pain.

*Impact*

My control over the management of my back pain is large.

I have a great deal of control over the management of my back pain.

I have considerable control over the management of my back pain.
